# Genome sequence of the clover symbiont *Rhizobium leguminosarum* bv. *trifolii* strain CC275e

**DOI:** 10.1186/s40793-015-0110-1

**Published:** 2015-12-08

**Authors:** Clément Delestre, Aurélie Laugraud, Hayley Ridgway, Clive Ronson, Maureen O’Callaghan, Brent Barrett, Ross Ballard, Andrew Griffiths, Sandra Young, Celine Blond, Emily Gerard, Steve Wakelin

**Affiliations:** University of Bordeaux, IT Science, 351 Cours de la Libération, 33400 Talence, France; AgResearch Ltd, Lincoln Campus, Private Bag 4749, Christchurch, 8140 New Zealand; Faculty of Agriculture and Life Sciences, Lincoln University, PO Box 84, Christchurch, New Zealand; Department of Microbiology and Immunology, University of Otago, PO Box 56, Dunedin, New Zealand; AgResearch Ltd, Grasslands Research Centre, Private Bag 11008, Palmerston North, New Zealand; South Australian Research and Development Institute, Urrbrae, South Australia Australia

**Keywords:** Root-nodule bacteria, Microsymbiont, Nitrogen fixation, Rhizobia, *Alphaproteobacteria*

## Abstract

*Rhizobium leguminosarum* bv. *trifolii* strain CC275e is a highly effective, N_2_-fixing microsymbiont of white clover (*Trifolium repens* L.). The bacterium has been widely used in both Australia and New Zealand as a clover seed inoculant and, as such, has delivered the equivalent of millions of dollars of nitrogen into these pastoral systems. *R. leguminosarum* strain CC275e is a rod-shaped, motile, Gram-negative, non-spore forming bacterium. The genome was sequenced on an Illumina MiSeq instrument using a 2 × 150 bp paired end library and assembled into 29 scaffolds. The genome size is 7,077,367 nucleotides, with a GC content of 60.9 %. The final, high-quality draft genome contains 6693 protein coding genes, close to 85 % of which were assigned to COG categories. This Whole Genome Shotgun project has been deposited at DDBJ/EMBL/GenBank under the accession JRXL00000000. The sequencing of this genome will enable identification of genetic traits associated with host compatibility and high N_2_ fixation characteristics in *Rhizobium leguminosarum.* The sequence will also be useful for development of strain-specific markers to assess factors associated with environmental fitness, competiveness for host nodule occupancy, and survival on legume seeds (New Zealand Ministry of Business, Innovation and Employment program, ‘Improving forage legume-rhizobia performance’ contract C10X1308 and DairyNZ Ltd.).

**Electronic supplementary material:**

The online version of this article (doi:10.1186/s40793-015-0110-1) contains supplementary material, which is available to authorized users.

## Introduction

White clover (*Trifolium repens*) is the most widely established and important legume in pastures in New Zealand [1] and globally [2]. In symbiosis with nodule-forming *Rhizobium leguminosarum* bacteria of the biovar *trifolii* (hereafter *R. leguminosarum**bv trifolii*), clover plants fix atmospheric nitrogen into a plant-available, thus providing an economically and environmentally sustainable method of maintaining soil fertility and pasture production. Across New Zealand there are 11,400+ farms using pastures containing forage legumes (mostly white clover), covering 7.88 million hectares [3]. This constitutes about 29 % of the total land area and excludes hill country/tussock grasslands. Estimates of nitrogen input from legumes vary, however average at 185 kg N ha^−1^ yr^−1^ for pastures with a slope less than 12° [4]. Based on recent average costs of urea fertilizer (2013–14 average), the value of N_2_ fixation into New Zealand pastures is 1.8 billion per year; this is highly conservative as it does not encompass the value of increased forage quality, N_2_ fixation in extensive hill country systems, and reduced environmental costs.

*R. leguminosarum**bv trifolii* strains vary extensively in their ability to form nodules with white clover [5], and also their effectiveness at fixing nitrogen during symbiosis [6]. As such, dedicated selection and screening programs have played a vital role in ensuring clover (and, of course, other legume species) are matched with an optimal rhizobia symbiont [7]. These are most commonly delivered into farming systems as rhizobia-inoculated seed [8].

The inoculation of white clover seed with rhizobia commenced in New Zealand in the early 20th century [8]. In addition to New Zealand produced inoculant strains, *R. leguminosarum**bv trifolii* strain CC275e was sourced from Australia [9]. From 1974, the inoculant production in New Zealand industry was phased-out and the sole commercial strain for inoculation of white clover seed was strain CC275e, which was then replaced with *R. leguminosarum**bv trifolii* strain TA1 (also from Australia) around 2005. Thus, *R. leguminosarum**bv trifolii* strain CC275e was in widespread use in New Zealand for approximately three decades, and is likely to have contributed billions of dollars of nitrogen into New Zealand’s pastoral systems. On white clover, *R. leguminosarum**bv trifolii* strain CC275e has been reported to fix more nitrogen than strain TA1 and has greater persistence in soils [9]. The decision by the inoculant industry to replace strain CC275e with strain TA1 was based on ease of production.

A number of synonyms of strain *R. leguminosarum**bv trifolii* strain CC275e exist. In New Zealand, a culture of strain CC275e was received by the Plant Diseases Division of the Department of Scientific and Industrial Research in 1974 and a re-isolate of this culture is referred to as strain PDD2163. Furthermore, in New Zealand, strain CC275e has also been referred to as strain W16 [10], but when used commercially was most commonly known as strain NZP561 [11]. In Australia, where the bacterium originates, early work referred to it as strain W16 or Strain Hastings T71 [10]. However, strain CC275e was the designation used when the bacterium was deposited in the CSIRO (Canberra) culture collection [12], and this is the most commonly used synonym. In the American Type Culture Collection, the bacterium is referred to as ATCC 35181. For this study, an original *R. leguminosarum**bv trifolii* strain CC275e culture was obtained from the Australian Inoculant Research Group (Gosford, NSW, Australia). These sequence data complements those of *Trifolium*-nodulating *R. leguminosarum**bv trifolii* strain WSM1325 (GenBank ID 241202755), strain WSM2304 (GenBank ID 209547612), strain WSM1689 (GenBank ID 752843554), and strain TA1 (GenBank ID 653806106).

## Organism information

### Classification and features

**Fig. 1:**
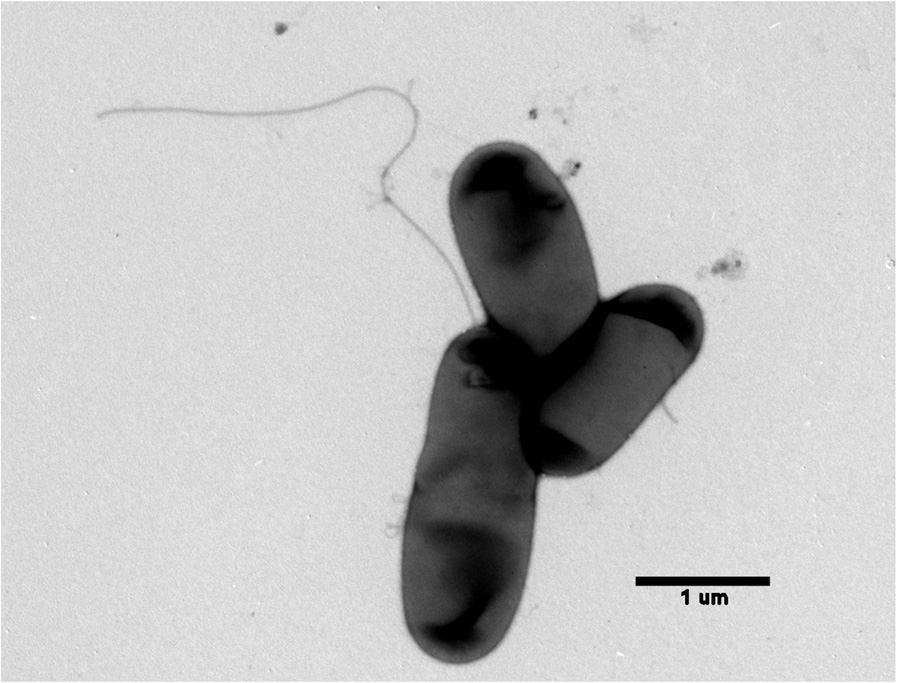
TEM micrograph of three *Rhizobium leguminosarum* bv. *trifolii* CC275e cells. The length of the bar = 1 um

*Rhizobium leguminosarum* bv. *trifolii* strain CC275e is a Gram-negative, motile, non-spore forming, non-encapsulated, rod shaped bacterium (Fig. 1). Colonies of *R. leguminosarum**bv trifolii* strain CC275e form within 4 to 5 days when grown on yeast mannitol agar (YMA; [13]) at 25 °C. Colonies are white-opaque, domed and glassy in appearance, with smooth margins.

*Rhizobium leguminosarum* and closely related species are generally regarded as non-fastidious, chemo-organotrophic bacteria [14]. Although the wider substrate requirements for strain CC275e have not been formally described, the authors support this classification based on personal experience in the handling, cultivation and fermentation of *R. leguminosarum**bv trifolii* strain CC275e.

**Fig. 2:**
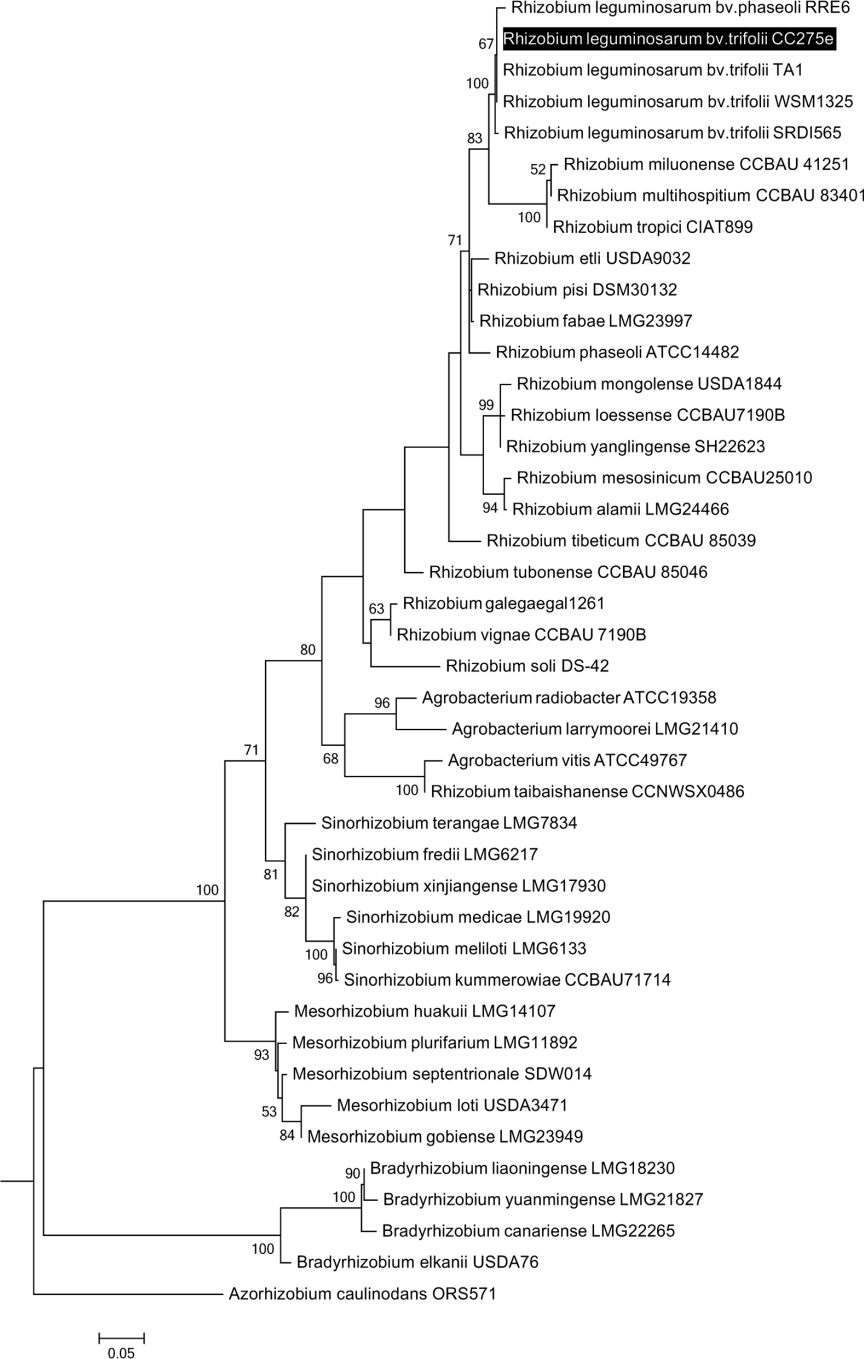
Phylogenetic tree showing relationship of *R. leguminosarum bv trifolii* CC275e with closely and distantly related taxa in the order *Rhizobiales*. The tree is based on 1498 bp length alignment of the 16S rRNA gene using MUSCLE with default parameters [31]. The tree was constructed using maximum likelihood method, with the General Time Reversible model (rate 4 classes; [32]). Nodes with bootstrap (1000 repetitions) support > 50 % are shown [33]. Accession numbers relating to the nucleotide sequences for each of the strains are listed in Additional file 1: Table S1

The *R. leguminosarum**bv trifolii* strain CC275e genome contains three (100 % identical) copies of the 16S rRNA gene. Alignment of these nucleotide sequences against other species supports close 16S rRNA phylogeny with *R. leguminosarum* originating from other legume hosts (Fig. 2). The 16S rRNA gene sequence has highest similarity to other accessions of *R. leguminosarum* biovars *trifolii* (99.8 %) and *phaseoli* (99.6 %) (Fig. 2) - the GenBank accession numbers for these are provided in Additional file 1: Table S1. The species is placed within the order *Rhizobiales* of the class *Alphaproteobacteria* [15]. Minimum information about the Genome Sequence (MIGS) is provided in Table 1.

**Table 1:** Classification and general features of *Rhizobium leguminosarum* bv. *trifolii* strain CC275e according to the MIGS recommendations [34]

MIGS ID	Property	Term	Evidence codes^a^
	Current classification	Domain *Bacteria*	TAS [35]
		Phylum *Proteobacteria*	TAS [36]
		Class *Alphaproteobacteria*	TAS [37]
		Order *Rhizobiales*	TAS [38]
		Family *Rhizobiaceae*	TAS [15]
		Genus *Rhizobium*	TAS [15]
		Species *Rhizobium leguminosarum*	TAS [14]
		Strain CC275e	TAS [12]
	Gram Stain	Negative	TAS [15]
	Cell Shape	Rod	TAS [15]
	Motility	Motile	TAS [15]
	Sporulation	Non spore-forming	TAS [15]
	Temperature range	Mesophile	TAS [15]
	Optimum temperature	28 °C	NAS
	pH range; optimum	Unknown	NAS
	Carbon source	Varied, chemoorganotrophic	TAS [15]
MIGS-6	Habitat	Soil, root nodule	TAS [12]
MIGS–6.3	Salinity	Non–halophile	NAS
MIGS–22	Oxygen requirement	Aerobic	TAS [15]
MIGS–15	Biotic relationship	Free living, legume symbiotic	TAS [15]
MIGS–14	Pathogenicity	Non–pathogen	TAS [15, 39]
MIGS–4	Geographic location	Tasmania, Australia	TAS [12]
MIGS–5	Sample collection date	1966	TAS [12]
MIGS–4.1	Latitude	Not recorded	
MIGS–4.2	Longitude	Not recorded	
MIGS–4.3	Depth	Not recorded	
MIGS–4.4	Altitude	Not recorded	

^a^Evidence codes – *IDA* Inferred from Direct Assay, *TAS* Traceable Author Statement (i.e., a direct report exists in the literature), *NAS* Non–traceable Author Statement (i.e., not directly observed for the living, isolated sample, but based on a generally accepted property for the species, or anecdotal evidence). These evidence codes are from the Gene Ontology project [34]

#### Symbiotaxonomy

*R. leguminosarum**bv trifolii* strain CC275e is nodule forming (Nod^+^) and N_2_ fixing (Fix^+^) on a range of annual and perennial clover host species. The original isolation of *R. leguminosarum**bv trifolii* strain CC275e was from *Trifolium repens* L. collected from Montague, North Western Tasmania [12], and has been used commercially due to its efficacy at forming symbioses and fixation of nitrogen on white clover hosts [9]. The strain is also moderately effective (*sensu* Brockwell et al. [12]) on *T. fragiferum* L. (strawberry clover; perennial), and *T. michelianum* Savi*,* (balansa clover; annual). On *T. subterraneum* L. (subterranean clover; annual), *T. purpureum* Lois. (purple clover; annual), and *T. hirtum* All. (rose clover; annual), strain CC275e has been described as effective [12].

## Genome sequencing information

### Genome project history

*R. leguminosarum**bv trifolii* strain CC275e was selected for sequencing based on its long history of commercial use as an inoculant for various clover (*Trifolium* spp.) hosts in Australia and New Zealand. In symbiosis with clover, this strain of bacteria has provided biologically-fixed nitrogen into soils for several decades, and thereby contributed to the fertility and productivity of pastoral agricultural systems in two countries. As part of a New Zealand MBIE-funded program, ‘Improving forage legume-rhizobia performance’ (C10X1308), the genomics of elite host nodulating (nod^+^) and N_2_ fixing (fix^+^) strains are being compared with closely related, ineffective strains. The aim is to identify markers to facilitate rhizobia selection programs, and to provide experimental tools for host colonization/competition experiments. Based on efforts in other *R. leguminosarum**bv trifolii* strains (see accessions listed in the introduction) a sequencing strategy was developed using a predicted genome size of approximately 7 Mb. The genome sequencing and assembly was completed in 2014; summary information on the project is given in Table 2. The final *R. leguminosarum**bv trifolii* CC275e genome assembly is a high-quality draft on 29 scaffolds, and resulted from approximately 150× sequencing coverage.

**Table 2:** Genome sequencing project information for *Rhizobium leguminosarum* bv. trifolii strain CC275e

MIGS ID	Property	Term
MIGS-31	Finishing quality	High-quality draft
MIGS-28	Libraries Used	Illumina TruSeq™ DNA Sample Preparation Kit V2, 2 × 150 bp paired end library
MIGS-29	Sequencing platform	Illumina MiSeq™
MIGS-31.2	Fold coverage	3.75 million reads, ≈150 × genome coverage
MIGS-30	Assemblers	A5, SSPACE, Velvet Optimiser
MIGS-32	Gene calling method	Glimmer 3
	Locus Tag	
	Genbank ID	JRXL00000000
	Genbank Date of Release	27st October, 2014
	GOLD ID	Gp0113226
	BIOPROJECT	259682
MIGS-13	Source Material Identifier	ATCC 35181
	Project relevance	Symbiotic N_2_ fixation, agriculture

### Growth conditions and genomic DNA preparation

A loop of a single colony of *R. leguminosarum**bv trifolii* CC275e was inoculated into YM broth [13] and grown to mid-log phase via incubation at 28 °C at 200 rpm for 12 h. DNA was extracted from the cell culture using a Gentra Puregene Cell kit (Qiagen). Spectrophotometry was used to quantify the DNA and ensure quality was sufficient for sequencing analysis (Nanodrop Thermo Scientific).

### Genome sequencing and assembly

Genome sequencing was conducted through NZGL (contract NZGL00940) at Massey University (MGS). Sequencing was performed on an Illumina MiSeq^TM^ instrument (details in Table 2), using 2 × 150 bp paired-end (PE) library with an average insert size of 420 bp. The sequencing run generated 3,751,285 reads totaling 1088 Mb of data.

Reads were assembled using the Java Assembling and Scaffolding Tool (JAST; [16]). Quality control of the sequence reads was conducted in Flexbar [17], and initial *de novo* assembly in A5 [18]; this resulted in 52 contigs. Bowtie2 [19] and Velvet [20] were further used to optimize the assembly, using the genome of the closely strain *R. leguminosarum* strain WSM1325 (Fig. 2) as a reference (NCBI accession 241202755). SSPACE [21] was used to assemble the 35 contigs into 29 scaffolds (Table 3). Summary details of the sequencing process are given in Table 2.

**Table 3:** Genome statistics for *Rhizobium leguminosarum* bv. *trifolii* strain CC275e

Attribute	Value	% of total
Genome size (bp)	7,077,367	100.00
DNA coding (bp)	6,201,447	87.62
DNA G + C (bp)	4,306,744	60.90
DNA scaffolds	29	
Total genes	6747	100.00
Protein coding genes	6693	99.00
RNA genes	54	0.80
Pseudo genes	not determined	not determined
Genes in internal clusters	not determined	not determined
Genes with function prediction	5018	74.37
Genes assigned to COGs	5722	84.80
Genes with Pfam domains	5682	84.22
Genes with signal peptides	531	7.87
Genes with transmembrane helices	1584	23.48
CRISPR repeats	0	

### Genome annotation

Annotation was added by the NCBI Prokaryotic Genome Annotation Pipeline (http://www.ncbi.nlm.nih.gov/genome/annotation_prok/). Clusters of orthologous groups of proteins (COGs) were predicted using COGnitor [22], and the presence of signal peptides was detected using SignalP [23]. Pfam domains were predicted using HMMER [24] against the Pfam-A database [25]. Transmembrane predictions and CRISPR repeats were found in Genious [26] using the Transmembrane prediction (http://www.geneious.com/plugins/transmembrane-prediction-plugin) and CRT plugins [27] respectively.

## Genome properties

The genome of *R. leguminosarum**bv trifolii* strain CC275e is estimated to be 7,077,367 nucleotides in size (Table 3). The GC content is 60.9 % which is similar to closely related strains such as *R. leguminosarum**bv trifolii* strain TA1 (60.74 %; [28]). The final draft consists of 29 scaffolds, the largest of which is 1,609,666 bp and the smallest 1167 bp. In total, 6747 genes were identified, 99 % of these were protein coding and the rest rRNA genes (Table 3). The majority of protein coding genes (84.22 %) have functionality predicted against COG categories; these are listed in Table 4. The remainder are listed as hypothetical.

**Table 4:** Number of protein coding genes of *Rhizobium leguminosarum* bv. *trifolii* strain CC275e associated with the general COG functional categories

Code	Value	% of total	COG category
J	189	2.69	Translation
A	0	0.00	RNA processing and modification
K	624	8.88	Transcription
L	186	2.65	Replication
B	2	0.03	Chromatin structure and dynamics
D	38	0.54	Cell cycle control
Y	0	0.00	Nuclear structure
V	64	0.91	Defense mechanisms
T	361	5.14	Signal transduction mechanisms
M	297	4.23	Cell wall/membrane/ biogenesis
N	96	1.37	Cell motility
Z	0	0.00	Cytoskeleton
W	0	0.00	Extracellular structures
U	74	1.05	Intracellular trafficking
O	185	2.63	Posttranslational modification
C	295	4.20	Energy production and conversion
G	646	9.19	Carbohydrate transport and metabolism
E	672	9.56	Amino acid transport and metabolism
F	108	1.54	Nucleotide transport and metabolism
H	151	2.15	Coenzyme transport and metabolism
I	238	3.39	Lipid transport and metabolism
P	234	3.33	Inorganic ion transport and metabolism
Q	95	1.35	Secondary metabolites biosynthesis
R	623	8.87	General function prediction only
S	544	7.74	Function unknown
-	1305	18.57	Not in COGs

**Fig. 3:**
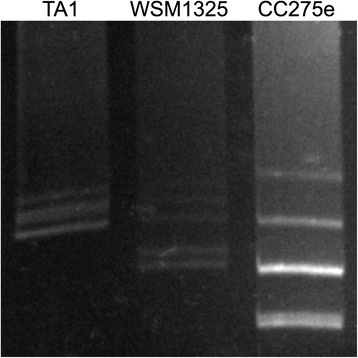
Eckhardt gel electropherogram showing ‘mega-plasmid’ profiles of *R. leguminosarum bv trifolii* strain CC275e against strains TA1 and WSM1325. The bright central band for strain CC275e represents co-migration of two similarly sized plasmids. Also, note double band at bottom of strain CC275e lane profile. The size of plasmids in reference strain WSM1325 are 294, 350, 516, and 829, 661, 516, 350, and 294 kb

Analysis of the genome by Eckhart gel electrophoresis [29] (Fig. 3) revealed the presence of six mega-plasmids. Mega-plasmids are typical of the ‘ancillary genome’ present in many *R. leguminosarum* strains [30] and commonly host many of the recognition factors associated with host compatibility, and nitrogen fixation. Based on the known mega-plasmid profile of *R. leguminosarum**bv trifolii* strain WSM1325 (Fig. 3), the mega-plasmids in *R. leguminosarum**bv trifolii* strain CC275e are approximately >1000, 500, 280, 280, 150, and 140 kb in size. As yet it is unknown to which scaffolds these mega-plasmids are associated.

## Conclusions

*Rhizobium**leguminosarium* bv. *trifolii* bacteria are an important resource for agricultural production [1, 2, 4]. In symbiosis with a suitable legume host (legume root nodules), atmospheric nitrogen fixed by these bacteria provides a source of plant nutrition that increases the farming system fertility in an economically and environmentally sustainable manner. Strains of *R. leguminosarum**bv trifolii* vary in host-compatibility between legume species [5], and their nitrogen fixation efficacy when in symbiosis [6]. Understanding the genetic factors controlling these, and other phenotypes such as saprophytic survival, and desiccation tolerance, will enable increased utilization of *R. leguminosarum**bv trifolii* for farming systems. The strain *R. leguminosarum**bv trifolii* strain CC275e has been commercially used as an inoculant for white-clover for several decades [9]. The genome sequencing of this ‘highly efficacious’ bacterium, allows for the identification of genetic factors associated with desirable phenotypes (see previous). This will be achieved by comparison of the *R. leguminosarum**bv trifolii* strain CC275e with closely related stains (e.g. based on 16S rRNA similarity) that differ in one or more phenotypes.

## Additional file

Additional file 1: Table S1. List of strain names and associated NCBI GenBank accession numbers for bacterial isolates in Fig. 2. (XLSX 13 kb)

## Abbreviations

CSIRO: Commonwealth scientific and industrial research organisation
Fix^+^: Nitrogen fixation positive
NZGL: New Zealand genomics Ltd
Nod^+^: Nodulation positive
MGS: Massey genome service
MBIE: Ministry of business, innovation and employment
*R. leguminosarum bv trifolii*: *Rhizobium leguminosarum* symbiovar *trifolii*
TEM: Transmission electron microscopy
YM: Yeast mannitol
